# Enhancing Water Splitting Performance via NiFeP-CoP on Cobalt Foam: Synergistic Effects and Structural Optimization

**DOI:** 10.3390/nano15120883

**Published:** 2025-06-07

**Authors:** Shihu Zhu, Yingxing Yang, Mengyao Zhao, Hui Zhao, Siyuan Liu, Jinyou Zheng

**Affiliations:** 1 State Key Laboratory of Coking Coal Resources Green Exploitation, Engineering Research Center of Advanced Functional Material Manufacturing of Ministry of Education, School of Chemical Engineering, Zhengzhou University, Zhengzhou 450001, China; 2 School of Mechanical and Power Engineering, Zhengzhou University, Zhengzhou 450001, China

**Keywords:** cobalt oxide hydroxide nanosheets, transition metal phosphide, oxygen evolution reaction, hydrogen evolution reaction, full water splitting

## Abstract

Hydrogen energy holds great promise for alleviating energy and environmental issues, with alkaline electrochemical water splitting being a key approach for hydrogen production. However, the high cost and limited availability of noble-metal catalysts hinder its widespread application. This study presents a novel method to fabricate a NiFeP-CoP/CF electrode. By growing CoOOH nanosheets on Co foam at low temperatures and filling the gaps between nanosheets with Ni and Fe phosphides, the prepared electrode exhibits outstanding electrocatalytic performance. For the oxygen evolution reaction (OER) in alkaline media, it requires overpotentials of only 235 mV and 290 mV to reach current densities of 10 mA cm^−2^ and 100 mA cm^−2^, respectively. In the case of the hydrogen evolution reaction (HER), overpotentials of 89 mV and 172 mV are needed to achieve current densities of −10 mA cm^−2^ and −100 mA cm^−2^. The NiFeP-CoP/CF-based electrolytic cell requires a cell voltage of only 1.70 V to achieve a current density of 100 mA cm^−2^ for overall water splitting. Moreover, during long-term continuous operation at 100 mA cm^−2^, the overpotential for OER remains constant while that for HER decreases. The low-temperature growth of CoOOH nanosheets on Co foam provides a new strategy for large-scale electrode production applicable in electrochemical processes and pollutant degradation. Significantly, filling the nanosheet gaps with phosphides effectively enhances the electrocatalytic performance of the system. This work offers a facile and cost-effective technique for the large-scale production of metallic (oxyhydr)hydroxides for electrocatalytic water splitting, showing great potential for industrial applications.

## 1. Introduction

Hydrogen energy offers a highly promising strategy for alleviating energy depletion and environmental pollution [1,2]. Currently, there is a growing global interest in hydrogen energy owing to its cleanliness and high mass-energy density [3,4,5]. For the generation of commercial high-purity hydrogen, alkaline electrochemical water splitting ranks among the most promising approaches [6,7,8]. Electrochemical water splitting consists of two half-cell reactions, namely the cathodic hydrogen evolution reaction (HER) and the anodic oxygen evolution reaction (OER) [9,10]. A number of catalysts, such as Pt, RuO_2_, IrO_2_, and other noble-metal-based electrocatalysts, have been explored for HER and OER, and these catalysts demonstrate excellent performance in either HER or OER [11,12,13,14]. Nevertheless, the utilization of noble metals has been significantly restricted due to their high cost and limited availability [15]. Therefore, researching non-noble metals with outstanding performance has become a top priority [16].

In recent research, the investigation into metal elements Fe, Co, and Ni, which possess strong electrocatalytic activity for the OER or/and HER in alkaline solutions, has attracted substantial attention [9,16,17,18,19,20,21,22,23,24]. For example, various cobalt-containing materials have been explored, such as cobalt oxides [25,26], cobalt (oxyhydr)oxides [27], cobalt nitrides [28,29], cobalt hydroxides [30], cobalt chalcogenides [31,32], cobalt phosphides [20,33,34,35,36,37,38,39], Co-N-C composites [40], Co-P-O composites, and so on [41]. These powder-type catalysts generally need to be combined with a polymer binder to fabricate electrodes. However, several factors reduce the efficiency of water splitting in such systems. Firstly, the specific surface area and the number of active sites of the powder-type catalysts can be diminished. This is because the polymer binder may cover some of the active sites, thereby reducing their accessibility to reactants [42]. Secondly, during the water splitting process, a large number of bubbles accumulate on the planar substrates. This accumulation impedes the mass and electron transfer between the electrolytes and the surface active sites, thus retarding the reaction kinetics [43,44]. Thirdly, the continuous generation of bubbles can cause the catalyst particles to be gradually detached from the electrodes. This not only decreases the effective surface area of the catalyst but also deteriorates the performance of the water splitting reaction [45,46]. Finally, the high-cost binder material and the complex binding process limit the practical application of these powder-type catalysts. In contrast, methods for fabricating thin electrode films on conductive surfaces using non-noble materials, such as hydrothermal reaction [47], electrodeposition [48], sputtering [49], and other techniques, are likely to be more effective than the traditional powder catalysts.

The interface between the substrate and the catalyst layer is significantly enhanced through the formation of thin films on conductive substrates. This improvement results in outstanding electrochemical water splitting performance, accompanied by long-term durability. A large number of combination electrodes were built utilizing Ni foam [50,51,52], carbon cloth [53,54,55], Cu foam [56,57,58], or other substrates. Co foam is extremely structurally robust, stable in alkaline solution during OER or HER. Recently, only a few studies using Co foam as a template for electrocatalytic water splitting have been reported. For instance, Xiong et al. [59] reported that one-step fabrication of Co_9_S_8_ particles supported on cobalt foam shows performance for OER with 350 mV to attain an anodic current density of 10 mA cm^−2^ and atomic-layer-deposited ultrafine MoS_2_ nanocrystals on cobalt foam with 270 mV to achieve an anodic current density of 10 mA cm^−2^ [60]. Unfortunately, most Co foam-based catalysts have lower electrocatalytic activity (HER and OER) than those of the noble-metal-based catalysts, regarding overpotential and long-term stability. Li et al. [61] reported bifunctional porous cobalt phosphide foam with high-current-density and long stability by electrochemical anodization, annealing, and phosphorization toward the HER with only 290 mV to achieve −1000 mA cm^−2^ and toward the OER with η = 380 mV to reach 1000 mA cm^−2^; a high temperature up to 500 °C gives rise to higher costs as well as a potential safety hazard. Additionally, fabricating a composite catalyst can serve as a highly effective strategy to enhance OER/HER performance [62,63]. Therefore, developing a Co foam-based composite catalyst that concurrently demonstrates excellent HER and OER capabilities in the alkaline electrolyte is urgently needed using a quick and cost-effective synthetic method.

Herein, a facile and cost-effective method for fabricating a NiFeP-CoP/CF electrode is reported. The fabrication process involves three main steps: alkalized treatment, phosphorization of Co foam, and loading of Ni-Fe phosphides. The stronger anchoring effect between the Co foam and the active NiFe-CoP catalyst significantly contributes to enhanced long-term stability. The addition of Ni-Fe phosphides further improves the HER performance and stability of the CoP catalyst. Comprehensive electrochemical tests and structure-performance analyses of NiFeP-CoP/CF are carried out to explore the structure–performance relationship, which is crucial for subsequent rational design.

## 2. Materials and Methods

### 2.1. Materials

Iron(III) chloride hexahydrate (FeCl_3_·6H_2_O, ≥99%), nickel(II) chloride hexahydrate (NiCl_2_·6H_2_O, ≥98%), sodium hydroxide (NaOH, ≥96%), and ethanol (C_2_H_5_OH, ≥99.7%) were purchased from Sinopharm Chemical Reagent Co., Ltd., Shanghai, China. Sodium hypophosphite monohydrate (NaH_2_PO_2_·H_2_O, ≥99%) was obtained from Shanghai Aladdin Bio-Chem Technology Co., Ltd., Shanghai, China. Sulfuric acid (H_2_SO_4_, 95~98%) was obtained from Luoyang Chemical Reagent Factory, Luoyang City, China. Co foam with an area density of 1400 g m^−2^ was purchased from Alantum Corporation, Seoul, Republic of Korea. All the chemicals were used without further purification. The pieces of Co foam (2.5 cm × 1.0 cm) were successively sonicated in anhydrous ethanol and 0.5 M H_2_SO_4_ solution for 10 min and 2 min to remove organic impurities and oxide layer, respectively, followed by washing with deionized (DI) water and ethanol, and finally dried by N_2_ flow for the next experiments.

### 2.2. Growing CoOOH Nanosheets on Co Foam (CoOOH/CF)

CoOOH/CF was synthesized by immersing Co foam in an alkaline solution followed by heating. First, the cleaned Co foam was soaked in a 4.0 M NaOH solution under sonication for 1 min. Subsequently, it was taken out and the surface solution was removed by a stream of N_2_. The NaOH-treated Co foam was then transferred into a glass bottle and heated at 80 °C for 12 h in an oven, resulting in the formation of a CoOOH film on the surface of the Co foam. After heating, the obtained CoOOH/CF was washed with DI water and ethanol, and finally dried by a stream of N_2_ for subsequent experiments.

### 2.3. Preparation of CoP Nanosheets on Co Foam (CoP/CF)

CoP/CF was fabricated through the phosphorization of CoOOH/CF in a tube furnace. Specifically, 1.0 g of NaH_2_PO_2_·H_2_O and the CoOOH/CF sample were placed separately in a quartz tube using two ceramic boats, with NaH_2_PO_2_·H_2_O positioned upstream. The quartz tube was purged with Ar gas for 30 min. Subsequently, under an Ar atmosphere, it was heated to 300 °C at a rate of 5 °C/min and held at this temperature for 1 h. After the system naturally cooled down to room temperature, CoP/CF was obtained. For the purpose of comparison, pure Co foam was directly phosphatized using the same procedure as that for CoP/CF, and the resulting sample was designated as Co-P/CF.

### 2.4. Fabrication of NiFeP on CoP/CF Nanoplates (NiFeP-CoP/CF)

NiFeP-CoP/CF was synthesized by loading Ni^2+^ and Fe^3+^ ions onto CoP/CF, followed by phosphorization. First, CoP/CF was immersed in a mixed solution containing 0.05 M NiCl_2_ and 0.05 M FeCl_3_. Then, it was taken out and heated at 80 °C for 4 h. This dipping–heating process was repeated, and the ion-loaded sample was subjected to the same phosphorization procedure as that for CoP/CF. For comparison, FeP-CoP/CF and NiP-CoP/CF were prepared by immersing CoP/CF in 0.1 M FeCl_3_ and 0.1 M NiCl_2_ solutions, respectively, followed by an identical phosphorization process. Additionally, the samples of NiFeP/Co, NiFeP/CoOOH, and NiFeP/Co-P were fabricated by immersing Co foam, CoOOH/CF, and Co-P/CF in the 0.05 M NiCl_2_ and 0.05 M FeCl_3_ mixed solution, respectively, and then applying the same phosphorization conditions.

### 2.5. Characterization

The morphology of the samples was characterized using scanning electron microscopy (SEM, FEI Nova NanoSEM 450, Billerica, MA, USA) operating at an accelerating voltage of 10 kV. Transmission electron microscopy (TEM, JEOL JEM-2100F, 200 kV, Tokyo, Japan) equipped with energy-dispersive spectroscopy (EDX, Oxford Instruments, INCA X-sight, High Wycombe, UK) was employed to investigate the crystallinity and elemental composition. The crystalline structure of the samples was determined by an X-ray diffractometer (Bruker D8 Advance, Karlsruhe, Germany, using Cu Kα radiation with λ = 1.54178 Å and a scan speed of 5 deg./min). The surface chemical environment was analyzed by X-ray photoelectron spectroscopy (XPS, Thermo ESCALAB 250XI, Carlsbad, CA, USA, with an Al Kα source gun). The obtained XPS spectra were calibrated with respect to the C 1s binding energy of 284.8 eV.

### 2.6. Electrochemical Measurements

All electrochemical tests were performed using a CHI660e electrochemical workstation (Shanghai Chenhua Instrument Co., Ltd., Shanghai, China) with a three-electrode configuration in a 1.0 M KOH solution (pH = 14). The as-prepared electrode served as the working electrode, and its geometric area was regulated to a 1 cm^2^ test area using insulating tape. A coiled platinum wire and a Ag/AgCl (in saturated KCl) electrode were employed as the counter and reference electrodes, respectively. Linear sweep voltammetry (LSV) curves were acquired at a scan rate of 1 mV s^−1^. All the potentials reported in this study were converted to the reversible hydrogen electrode (RHE) scale according to the following equation [64,65]. E_RHE_ = E_Ag/AgCl_ + E^0^_Ag/AgCl_ + 0.0591 × pH, where E_Ag/AgCl_ is the experimental potential measured with respect to the Ag/AgCl (in saturated KCl) reference electrode.

The photovoltaic water electrolysis application was realized through the fabrication of a system comprising NiFeP-CoP/CF electrodes (1 × 1 cm^2^) as the anode and cathode, which were integrated into a water-splitting reactor. The reactor was electrically connected to a crystalline silicon solar cell with a rated voltage of 1.5 V and a rated current of 435 mA. The solar cell had dimensions of 60 × 80 cm and a peak power output of 0.65 W. The electrolyte used for water splitting was 1.0 M KOH.

## 3. Results and Discussion

**Figure 1:**
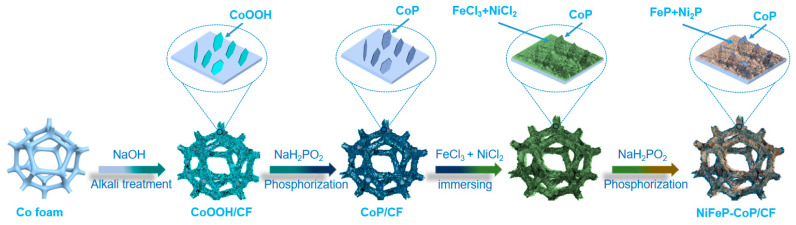
Schematic illustration of procedure for fabrication of NiFeP-CoP/CF electrode.

As depicted in Figure 1, the NiFeP-CoP/CF was synthesized through a straightforward three-step procedure involving alkali treatment, phosphorization, immersion, and re-phosphorization. Owing to its excellent structural properties in an alkaline solution, commercial Co foam was selected as the substrate for film fabrication. Initially, hexagonal CoOOH nanosheets could be in situ generated by simply treating and heating the commercial Co foam. The Co foam was immersed in a 4.0 M NaOH solution and then promptly removed. A very thin layer of the NaOH solution was uniformly distributed throughout most of the macropores, and the excess solution on the surface was blown off by a stream of N₂. In the subsequent heating process, the oxygen in the air served as an oxidant. Other reaction conditions, such as the remaining NaOH and the relatively low heating temperature of 80 °C, accelerated the oxidation process and promoted corrosion. Since oxyhydroxide is more amenable to phosphorization than pure metal at low temperatures, the preparation of CoOOH/CF is crucial as it facilitates the phosphorization process (Figures S1 and S2). The phosphorization of CoOOH/CF (designated as CoP/CF) increased the number of active sites and the surface roughness of the nanosheets, both of which were vital for enhancing the electrocatalytic hydrogen evolution reaction (HER) performance. Moreover, the rough surfaces enabled the adhesion of Ni and Fe ions to each other in the subsequent step. After CoP/CF was immersed in a mixed solution, heated at 80 °C, and phosphorized at 300 °C, FeP and Ni_2_P were ultimately filled into the gaps of the nanosheets.

**Figure 2:**
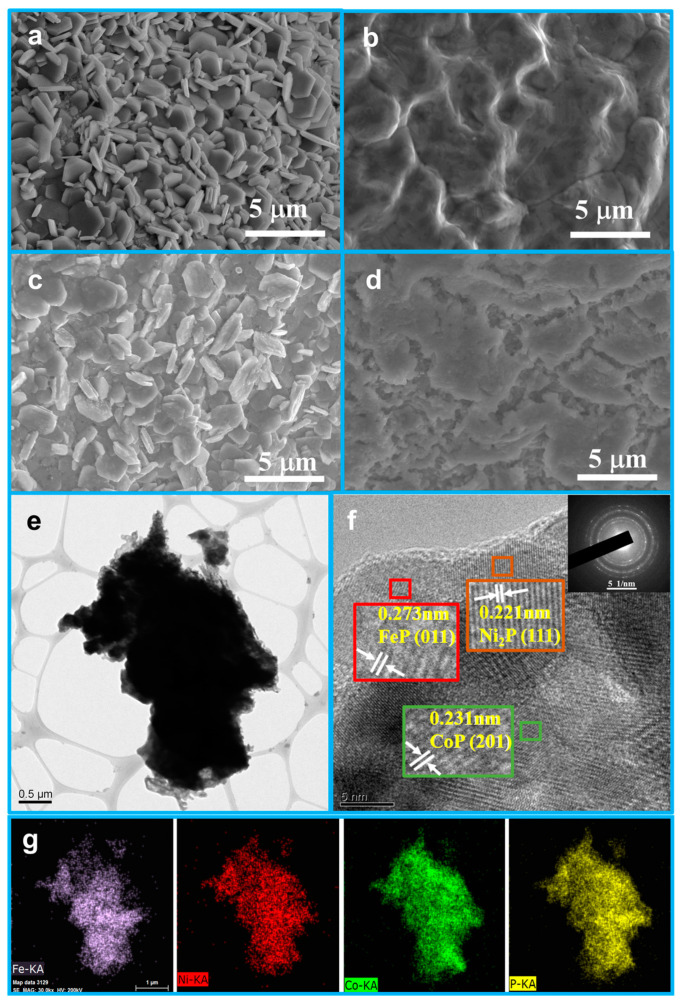
SEM images of (**a**) CoOOH/CF, (**b**) Co-P/CF, (**c**) CoP/CF, and (**d**) NiFeP-CoP/CF. (**e**) TEM, (**f**) HRTEM, and (**g**) EDS mapping images of NiFeP-CoP/CF. The inset in (**f**) is the corresponding SAED pattern.

The morphologies of the CoOOH/CF, Co-P/CF, CoP/CF, and NiFeP-CoP/CF samples were observed via SEM images, as presented in Figure 2. After undergoing alkali and heat treatments, the surface of the Co foam was transformed into CoOOH/CF, as shown in Figure 2a. The CoOOH/CF exhibits hexagonal nanosheets that are dense, possess a smooth surface, and are uniform in size. Subsequently, the CoP/CF shown in Figure 2c was obtained through phosphorization. This process altered the surface roughness of the nanosheets while maintaining their hexagonal structure. To compare the effects of different treatments and demonstrate how alkali treatment influenced the subsequent phosphorization process, direct phosphorization was carried out on both CoOOH/CF and Co foam. In contrast to CoP/CF, the directly phosphorized Co foam (the Co-P/CF sample) formed a surface similar to that of pure Co foam in terms of smoothness and the absence of obvious nanoparticles (Figure S1). Evidently, the heating and immersion steps have a substantial impact on the subsequent phosphorization process. Finally, after the loading of transition metals (Figure S3) and re-phosphorization (Figure 2d), Fe^3+^ and Ni^2+^ precursors were partially filled into the gaps of the nanosheets, resulting in a surface that tended to be smoother. Furthermore, the microstructure information of NiFeP-CoP/CF was determined using TEM. Both the TEM image (Figure 2e) and the EDX elemental mapping (Figure 2g) indicate that Fe, Ni, Co, and P elements are uniformly distributed. According to the TEM analysis, the lattice spacing of the particles are approximately 0.273 nm, 0.221 nm, and 0.231 nm, which are consistent with the (011) planes of the FeP crystal, the (111) planes of the Ni_2_P crystal, and the (201) planes of the CoP crystal, respectively (Figure 2f). The ring-shaped diffraction pattern of the selected-area electron diffraction (SAED) (Figure 2f) reveals that the particle has a polycrystalline structure.

**Figure 3:**
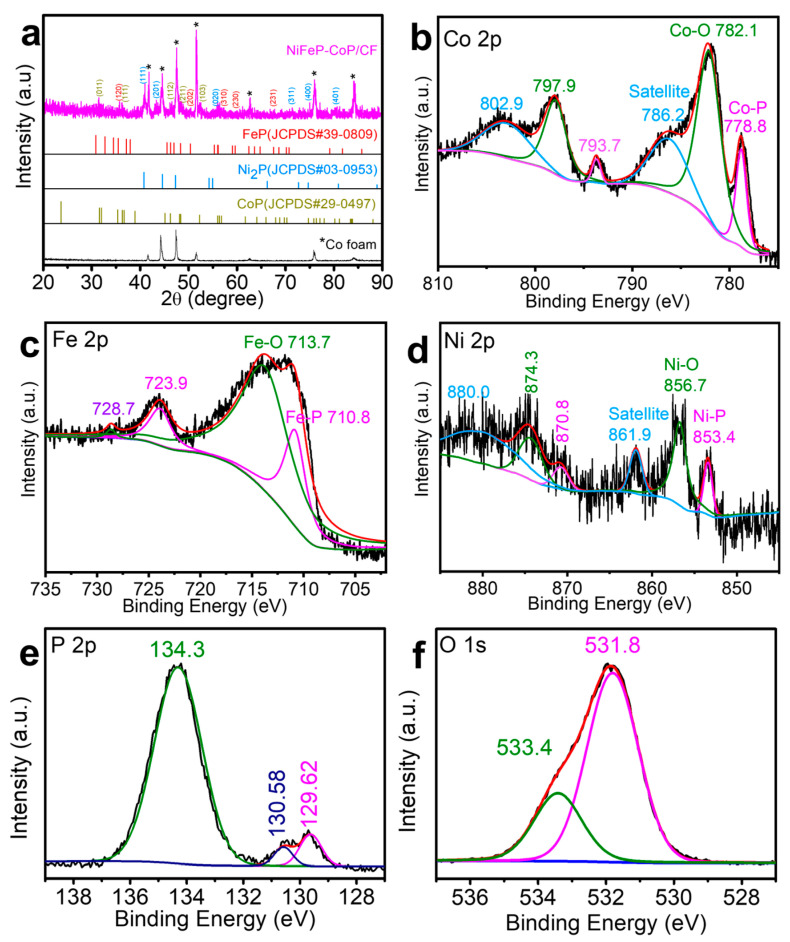
XRD and XPS patterns of the NiFeP-CoP/CF. (**a**) XRD pattern. XPS spectra of (**b**) Co 2p, (**c**) Fe 2p, (**d**) Ni 2p, (**e**) P 2p, and (**f**) O 1s element.

The crystal structure of CoOOH/CF and NiFeP-CoP/CF were verified by XRD, as depicted in Figure S4 and Figure 3a. Both XRD patterns indicated that the Co foam was not completely transformed into CoOOH or CoP, and the Co peak from Co foam persisted throughout the entire process. Additionally, new XRD peaks for CoOOH at 2θ angles of 20.2°, 37.0°, 38.9°, 45.9°, 50.6°, and 65.0° were observed. These peaks corresponded to the (003), (101), (012), (104), (015), and (110) planes of hexagonal CoOOH (JCPDS No. 07-0169) (Figure S4) [66]. As can be observed in Figure 3a, besides the seven characteristic peaks at 41.7°, 44.2°, 47.6°, 51.5°, 62.7°, 76.0°, and 84.2° that were consistent with Co foam, there were additional peaks corresponding to FeP (JCPDS No. 39-0809) [67], Ni_2_P (JCPDS No.03-0953) [68], and CoP (JCPDS No.29-0497) [67]. The peak of CoOOH disappeared after phosphorization, confirming the complete transformation of CoOOH into CoP. These findings further demonstrated that alkali treatment facilitated the phosphorization process and suggested the presence of Co, Ni, and Fe phosphides.

The XPS provided information on the precise chemical states of the Fe, Co, Ni, P, and O elements in NiFeP-CoP/CF. According to Figure 3b, the Co 2p_3/2_ and Co 2p_1/2_ peaks of CoP located at 778.8 eV and 793.7 eV, respectively, and their satellite peaks at 786.2 eV and 802.9 eV were attributed to surface oxidation [38]. As shown in Figure 3c, the binding energies at 710.8 eV and 723.9 eV were assigned to Fe 2p_3/2_ and Fe 2p_1/2_ of FeP, while 713.7 eV and 728.7 eV were oxide peaks. Similarly, for Ni_2_P, the peaks were observed at 853.4 eV for Ni 2p_3/2_, 870.8 eV for Ni 2p_1/2_, with 856.7 eV and 874.3 eV corresponding to oxide peaks, and 861.9 eV and 880 eV appearing as satellite peaks (Figure 3d) [69]. Additionally, as shown in Figure 3e, P 2p_1/2_ and P 2p_3/2_ were located at 129.6 eV and 130.6 eV, and the peak of 134.3 eV was attributed to metal phosphides (metal-P) and oxidized P species (P-O) [70]. The O 1s peaks at 531.8 eV and 533.4 eV corresponded to phosphate radical and adsorbed H_2_O as shown in Figure 3f [69,71]. Due to the contact between metal phosphide and air, an oxidation reaction occurred, leading to the formation of metal-based oxide peaks in NiFeP-CoP/CF. This emphasizes the presence of Ni^2+^, Co^2+^, and Fe^3+^ in NiFeP-CoP/CF, which is in agreement with the TEM results.

**Figure 4:**
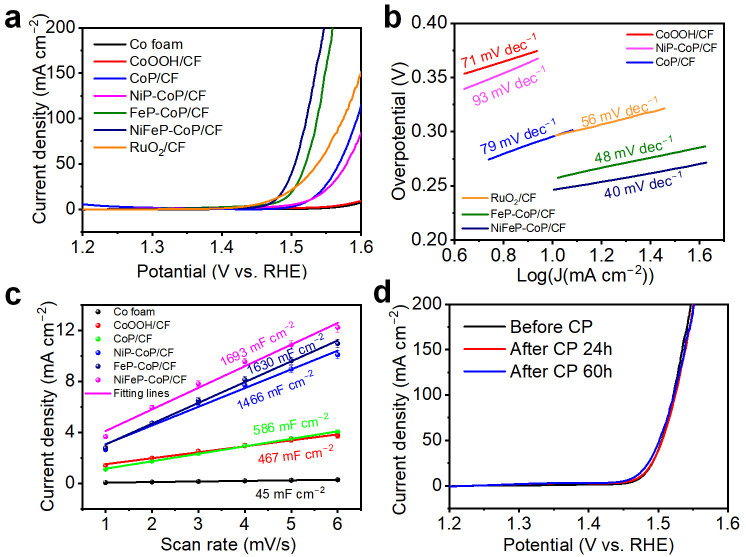
Evaluation of alkaline OER performance in 1.0 M KOH solution. (**a**) Polarization curves and (**b**) Tafel plots of CoOOH/CF, CoP/CF, NiP-CoP/CF, FeP-CoP/CF, NiFeP-CoP/CF, and RuO_2_/CF electrodes. (**c**) C_dl_ values of cobalt foam, CoOOH/CF, CoP/CF, NiP-CoP/CF, FeP-CoP/CF, and NiFeP-CoP/CF electrodes. The experimental data points are the average values of three independent tests. (**d**) Polarization curves of the as-obtained NiFeP-CoP/CF before and after chronopotentiometry measurement at a current density of 100 mA cm^−2^ for 24 h and 60 h.

A three-electrode system was employed to evaluate the OER performances of the electrodes in a 1.0 M KOH alkaline solution. All electrochemical measurements were conducted without stirring. As shown in Figure 4a, the linear sweep voltammetry (LSV) curves of all samples were measured at a slow scan rate of 1 mV s^−1^ to minimize the impact of non-faradaic current. The CoP/CF electrode requires overpotentials of only 295 mV and 367 mV to achieve current densities of 10 mA cm^−2^ and 100 mA cm^−2^ for OER, respectively, as shown in Table S1. These values are smaller than those of the Co foam, CoOOH/CF, and the electrode obtained by direct phosphorization of Co foam (Co-P/CF) as shown in Figure 4a and Figure S6a.

Considering the challenges associated with the phosphorization of Co and the reduced surface area of Co-P/CF compared to the CoP/CF electrode, the Co-P/CF electrode exhibits a relatively large OER overpotential. As demonstrated in Figure 4a, the NiFeP-CoP/CF electrode shows low overpotentials of 235 mV and 290 mV to achieve current densities of 10 mA cm^−2^ and 100 mA cm^−2^, respectively. This performance is even superior to that of the RuO_2_ catalyst (240 mV for 10 mA cm^−2^ and 345 mV for 100 mA cm^−2^). As is evident from Figure 4a and Figure S9a, the NiFeP-CoP/CF electrode performs the best when compared with Ni_2_P-CoP/CF, FeP-CoP/CF, and CoP-P/CF (CoP/CF is re-phosphorized). A possible explanation is that for Ni_2_P-CoP/CF, the loaded Ni_2_P, and for CoP-P/CF, the re-phosphorization of CoP, both decrease the electrical conductivity, and the formed phosphide covers part of the active sites, thus having a negative effect on OER. Although for FeP-CoP/CF, the loaded FeP, and for NiFeP-CoP/CF, the loaded Ni_2_P and FeP, fill the spaces between nanosheets, reducing their specific surface area, FeP can provide more active sites and induce a synergistic effect between FeP and CoP on the electrode surface. Additionally, for NiFeP-CoP/CF, another synergistic interaction between FeP and Ni_2_P is formed, and the Fe element can lower the average oxidation state of Ni [72]. These findings suggest that the OER overpotential can be reduced by filling the spaces in the CoP hexagonal nanosheets with FeP and Ni_2_P.

When elucidating the catalytic kinetics and mechanism, the Tafel slope is widely regarded as a pivotal kinetic parameter. As illustrated in Figure 4b, the NiFeP-CoP/CF electrode exhibits a Tafel slope of 40 mV dec^−1^, which is notably smaller than those of the NiP-CoP/CF (93 mV dec^−1^), CoP/CF (79 mV dec^−1^), CoOOH/CF (71 mV dec^−1^), RuO_2_/CF (56 mV dec^−1^), and FeP-CoP/CF (48 mV dec^−1^) electrodes. This observation implies that the NiFeP-CoP/CF electrode sustains a faster kinetic process for the OER. To assess the intrinsic activity of the synthesized catalysts, the double-layer capacitance (C_dl_) was measured, as it is directly proportional to the electrochemically active surface areas (ECSA) [73]. As depicted in Figure S11, where cyclic voltammetry (CV) curves at different scan rates within a non-faradic potential range were obtained, the NiFeP-CoP/CF electrode demonstrates a significantly larger C_dl_ value (846 mF cm^−2^) compared to those of CF (23 mF cm^−2^), CoOOH/CF (234 mF cm^−2^), CoP/CF (293 mF cm^−2^), NiP-CoP/CF (733 mF cm^−2^), and FeP-CoP/CF (815 mF cm^−2^) electrodes. This suggests that the NiFeP-CoP/CF electrode possesses a larger ECSA than the other electrodes, as clearly shown in Figure 4c. Based on the results of the LSV measurements, it can be inferred that the formation of CoP hexagonal nanosheets, along with the filling of Fe and Ni phosphides within the interstices of these nanosheets, contributes to a higher ECSA for the electrode. To evaluate the electrochemical stability of the electrodes for OER, the polarization curves were examined following the chronopotentiometry (CP) stability test at a current density of 100 mA cm^−2^. As depicted in Figure 4d, the LSV curves of the NiFeP-CoP/CF electrode nearly overlap after continuous operation for 0 h, 24 h, and 60 h. The CP curve of the NiFeP-CoP/CF electrode shows minimal fluctuations during 24 h continuous operation (Figure S14a). This indicates the remarkable stability of the NiFeP-CoP/CF electrode for OER in an alkaline solution.

The CoP/CF electrode, which features a hexagonal CoP nanosheet structure without phosphide filling, exhibits extremely steep LSV curves and a sharply increasing potential during the CP examination (Figure S7a,b). These results demonstrate that filling the gaps between CoP nanosheets with FeP and Ni_2_P can enhance the long-term electrochemical stability at high current densities. Comparison experiments involving different electrodes for OER testing were also conducted (Figures S10a–f and S14a). The loaded Ni2P and FeP on Co foam or Co-P/CF are prone to peeling off from the smooth surface of the substrate during the CP process. The LSV curves before and after 24 and 60 h CP operation (Figure S10d,e) reveal a significant decline in the OER performance of the NiFeP/Co and NiFeP/Co-P electrodes. Their overpotentials exceed those of the NiFeP-CoP/CF electrode after 24 h and 60 h of CP measurement (Figure S10a–c). Although the NiFeP/CoOOH electrode shows relatively stable performance before and after 24 h and 60 h of CP (Figure S10f), its CP curve displays substantial fluctuations throughout the CP test (Figure S14a), and its overpotential remains higher than that of the NiFeP-CoP/CF electrode throughout the CP testing period (Figure S10a–c). Overall, the NiFeP/CoOOH electrode performs worse than the NiFeP-CoP/CF electrode in terms of OER. To improve electrochemical durability, it is proposed that constructing a hexagonal CoP nanosheet structure and filling the nanosheet gaps with Ni and Fe phosphides are of great significance.

**Figure 5:**
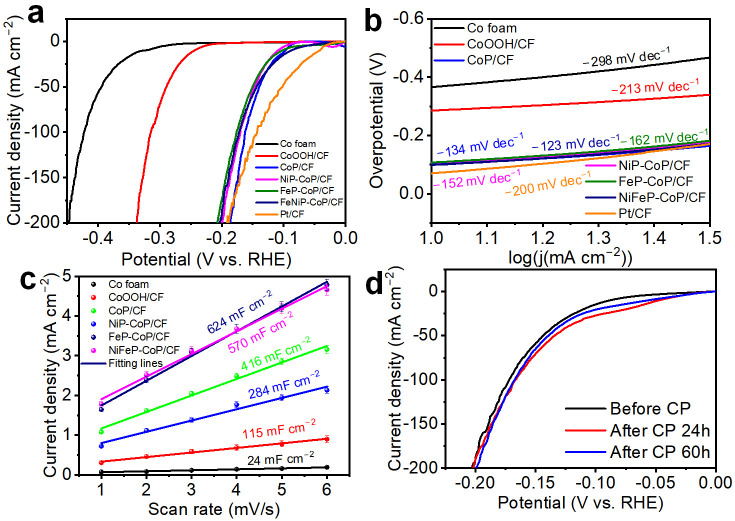
Evaluation of alkaline HER performance in 1.0 M KOH solution. (**a**) Polarization curves and (**b**) Tafel plots of cobalt foam, CoOOH/CF, CoP/CF, NiP-CoP/CF, FeP-CoP/CF, NiFeP-CoP/CF, and Pt-C/CF electrodes. (**c**) C_dl_ values of cobalt foam, CoOOH/CF, CoP/CF, NiP-CoP/CF, FeP-CoP/CF, and NiFeP-CoP/CF electrodes. The experimental data points are the average values of three independent tests. (**d**) Polarization curves of the as-obtained NiFeP-CoP/CF before and after chronopotentiometry measurement at a current density of −100 mA cm^−2^ for 24 h and 60 h.

To assess the performance of the electrodes in an alkaline HER test, relevant experiments were conducted in a 1.0 M KOH solution. The CoP/CF electrode, with its hexagonal CoP nanosheets, requires overpotentials of only 98 mV and 163 mV to reach current densities of −10 mA cm^−2^ and −100 mA cm^−2^, respectively (Figure S6b). These values are lower than those of the Co foam and Co-P/CF electrodes as shown in Tables S2. The large specific surface area of the hexagonal nanosheet structure and the excellent HER performance of CoP significantly reduce the HER overpotential of the electrodes. As depicted in Figure 5a, the loading of FeP or Ni_2_P does not seem to have any favorable effects on the HER overpotential. At relatively high current densities, the NiP-CoP/CF, FeP-CoP/CF, and NiFeP-CoP/CF electrodes essentially converge at a current density of 200 mA cm^−2^, which lies between that of CoP/CF and the noble-metal catalyst Pt-C/CF. Given that the NiFeP-CoP/CF electrode exhibits better OER performance than NiP-CoP/CF and FeP-CoP/CF, it could potentially be applied for HER and integrated for overall water splitting. The overpotential increased slightly when phosphide was added to the surface of the CoP/CF electrode. This is because the additional active sites generated by the phosphide addition are insufficient to counterbalance the negative impacts of the reduced contact area between the catalyst surface and the electrolyte and the slight loss of electrical conductivity. During the HER experiment, these phosphides rarely exerted a synergistic effect on each other. However, as revealed by the subsequent electrochemical durability test, the NiFeP-CoP/CF electrode is more stable than the CoP/CF electrode. Filling the gaps between the nanosheets enhances the overall HER performance.

According to Figure 5b, the NiFeP-CoP/CF electrode exhibits a Tafel slope of only 123 mV dec^−1^, which is lower than that of the precious-metal Pt-C/CF electrode (200 mV dec^−1^) and other control samples. This indicates that the NiFeP-CoP/CF electrode has a faster kinetic process during HER. To evaluate the intrinsic HER activity of the synthesized catalysts, the C_dl_ was also measured, as shown in Figure 5c. The highest C_dl_ value of NiFeP-CoP/CF, along with the electrochemical impedance spectroscopy (EIS) results in Figure S15, demonstrate that it has a large electrochemically active surface area and the lowest charge-transfer resistance. This highlights the advantage of forming hexagonal nanosheets and filling the gaps with phosphides in enhancing the electrochemically active surface area and reducing charge-transfer resistance. To verify the role of constructing a hexagonal nanosheet structure and filling the nanosheet gaps in terms of stability during the HER process, we selected various control samples and evaluated their long-term electrochemical stability by chronopotentiometry at a current density of −100 mA cm^−2^. As shown in Figure 5d, the LSV curve of NiFeP-CoP/CF remained nearly unchanged after continuous operation for 0 h, 24 h, and 60 h. The CP curve also showed some fluctuations during 24 h continuous operation (Figure S14b). The CoP/CF electrode with the hexagonal CoP nanosheet structure but without gap-filling had the lowest initial overpotential. However, its LSV curve exhibited significant degradation, and the potential in the CP test decreased substantially during the CP testing (Figure S7c,d). HER control tests with different electrodes were also carried out (Figures S6b, S9b and S12a–c). Initially, the overpotentials of electrodes with Ni_2_P and FeP loaded on Co foam or Co-P/CF were higher than those of NiFeP-CoP/CF and NiFeP/CoOOH (Figure S12b). After 24 h of CP testing, the overpotential of NiFeP/CoOOH changed from being initially lower than that of NiFeP-CoP/CF to being higher (Figure S12a,c). In conclusion, the NiFeP-CoP/CF electrode, as an excellent catalyst, indicates that fabricating a hexagonal CoP nanosheet structure and filling the gaps with Ni and Fe phosphides can simultaneously enhance the HER performance.

**Figure 6:**
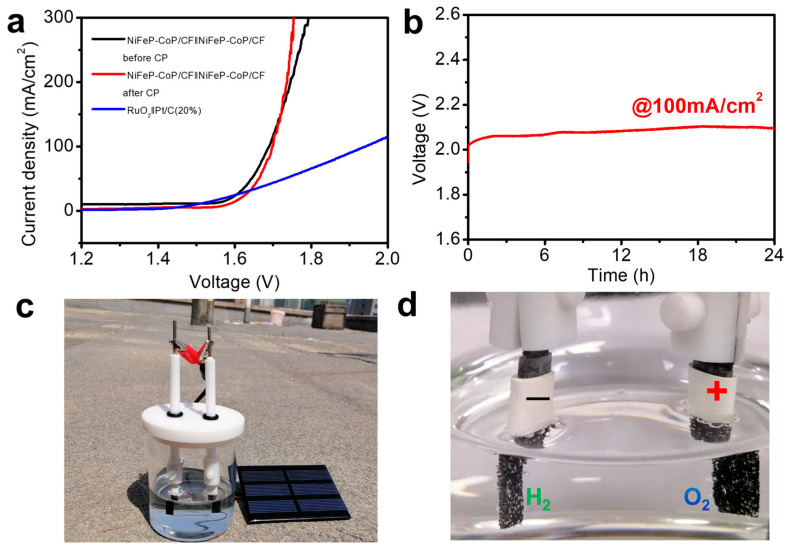
(**a**) Polarization curves of the NiFeP-CoP/CF couple and RuO_2_/CF||Pt/C/CF in 1.0 M KOH solution for overall water splitting. (**b**) Long-term stability test measured at a constant current density of 100 mA cm^−2^. (**c**,**d**) A photographic image of the demonstration of a 1.5 V solar panel powered for overall water splitting.

**Figure 7:**
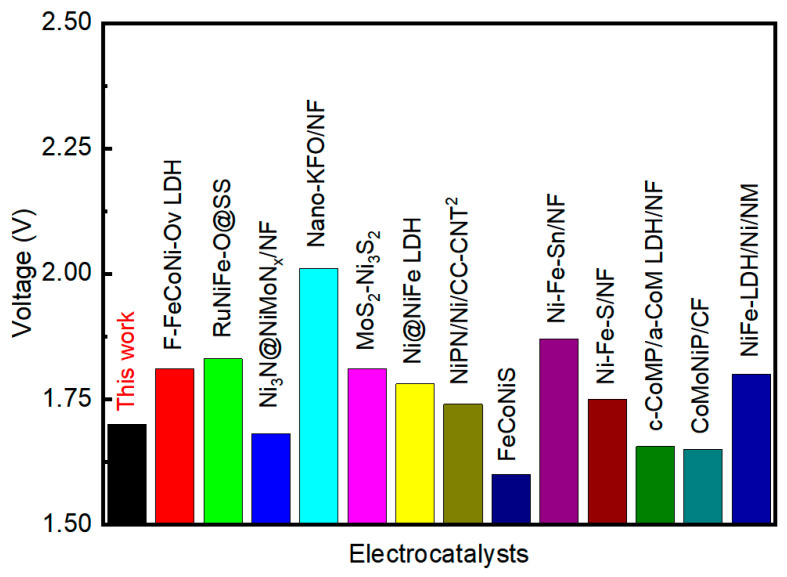
A comparison of the water splitting performance of the catalyst in this work with other reported bifunctional catalysts [74,75,76,77,78,79,80,81,82,83,84,85,86] at 100 mA cm^−2^.

The results demonstrate that the NiFeP-CoP/CF electrode simultaneously exhibits the best performance in both the OER and the HER. Given its low overpotential and high stability, NiFeP-CoP/CF was directly utilized in a two-electrode system for overall water splitting in an alkaline solution. As depicted in Figure 6a, the NiFeP-CoP/CF based electrolytic cell requires a cell voltage of only 1.70 V to achieve a current density of 100 mA cm^−2^. The experimental results reveal that after 24 h CP measurement, the overpotential decreases at current densities above approximately 150 mA cm^−2^. This indicates that high current densities are favorable for the operation of NiFeP-CoP/CF. As shown in Figure 6b,c, a 1.5 V solar panel was used to drive the water splitting process. Bubbles of oxygen and hydrogen were observed to continuously form on the surfaces of the anode and cathode, respectively. A comparison of the overall water splitting performance of NiFeP-CoP/CF with that of some previously reported typical electrocatalysts is presented in Figure 7, highlighting the strong bifunctional electrocatalytic performance of this material. In conclusion, NiFeP-CoP/CF is an efficient catalyst for overall water splitting. Its excellent performance suggests great potential for industrial applications.

## 4. Conclusions

In conclusion, this work has revealed that the NiFeP-CoP/CF electrode, fabricated by growing CoOOH nanosheets on Co foam at low temperatures and filling the gaps between nanosheets, demonstrates a low overpotential and excellent long-term stability for both the oxygen evolution reaction (OER) and the hydrogen evolution reaction (HER). For the alkaline OER, it requires overpotentials of only 235 mV and 290 mV to reach current densities of 10 mA cm^−2^ and 100 mA cm^−2^, respectively. In the case of HER, overpotentials of 89 mV and 172 mV are needed to achieve current densities of −10 mA cm^−2^ and −100 mA cm^−2^, respectively. During long-term continuous operation at a current density of 100 mA cm^−2^, the overpotential for OER remains constant, while that for HER decreases. Moreover, the low-temperature rapid growth of CoOOH nanosheets on Co foam offers a novel strategy for the large-scale production of electrodes applicable in electrochemical processes and pollutant degradation. Significantly, the electrocatalytic performance of the nanosheets can be enhanced by effectively filling the gaps with phosphides.

## Data Availability

The data presented in this study are available on request from the corresponding author.

## Supplementary Materials

The following supporting information can be downloaded at: https://www.mdpi.com/article/10.3390/nano15120883/s1, Figure S1: SEM image of the bare Co foam; Figure S2: XRD patterns of Co-P and Co foam; Figure S3: SEM image of NiFe-CoP/CF; Figure S4: XRD patterns of CoOOH/CF and Co foam; Figure S5: OER and HER polarization curves of the different CoP/CF electrodes obtained from CoOOH with different growing times of 0.5 h, 12 h, and 24 h in 4 M NaOH solution; Figure S6: OER and HER polarization curves of Co foam, CoOOH/CF, CoP/CF, and Co-P/CF in 1.0 M KOH solution; Figure S7: Long-term tests of CoP/CF at 100 mA/cm^2^. OER and HER polarization curves of CoP/CF, chronopotentiometry measurements of OER and HER at a current density of 100 mA/cm^2^ for 24 h; Figure S8: OER polarization curves of NiFeP-CoP/CF electrodes obtained from immerging in a solution containing 0.01 M (0.005 M NiCl_2_ and 0.005 M FeCl_3_), 0.1 M (0.05 M NiCl_2_ and 0.05 M FeCl_3_), and 1.0 M (0.5 M NiCl_2_ and 0.5 M FeCl_3_) mixed Fe^3+^ and Ni^2+^. OER polarization curves of NiFeP-CoP/CF obtained from immerging in a solution containing 1.0 M (0.5 M NiCl_2_ and 0.5 M FeCl_3_) mixed Fe^3+^ and Ni^2+^ solution before and after CP measurement at 100 mA/cm^2^; Figure S9: OER and HER polarization curves of CoP/CF, CoP-P/CF (phosphorization of CoP/CF); Figure S10: Long-term OER stability testing of NiFeP/Co, NiFeP/CoOOH, NiFeP-CoP/CF, and NiFeP/Co-P at 100 mA/cm^2^ for 24 h and 60 h; Figure S11: CV curves showing the capacitive of electrochemical double layer of Co foam, CoOOH/CF, CoP/CF, NiP-CoP/CF, FeP-CoP/CF, and NiFeP-CoP/CF for OER; Figure S12: Long-term HER stability testing at 100 mA/cm^2^ for 24 h and 60 h. HER Polarization curves of NiFeP/CoOOH, after CP measurement, before and after CP measurements; Figure S13: CV curves showing the capacitive of electrochemical double layer of Co foam, CoOOH/CF, CoP/CF, NiP-CoP/CF, FeP-CoP/CF, and NiFeP-CoP/CF for HER; Figure S14: Long-term OER and HER stability testing by chronopotentiometry measurement at 100 mA/cm^2^ for 24 h; Figure S15: Electrochemical impedance spectroscopy (EIS) of electrodes were testing with 0.01 Hz–100 kHz for OER and HER. Tables S1 and S2: The overpotentials of different samples for OER and HER.
