# Supplementary material for: Enhancing Water Splitting Performance via NiFeP-CoP on Cobalt Foam: Synergistic Effects and Structural Optimization
Source: Nanomaterials (Basel). 2025 Jun 7;15(12):883. doi: 10.3390/nano15120883 (PMC12196238; doi:10.3390/nano15120883)
Supplement: Supplementary file 1 [file nanomaterials-15-00883-s001.zip › nanomaterials-3616786-supplementary.pdf]

Supplementary Materials

Enhancing Water Splitting Performance via NiFeP-CoP on Cobalt Foam: Synergistic Effects and Structural Optimization

Shihu Zhu [1,2], Yingxing Yang [1], Mengyao Zhao [1], Hui Zhao [1], Siyuan Liu [1] and Jin You Zheng [1,*]

[1] *State Key Laboratory of Coking Coal Resources Green Exploitation at Zhengzhou University; Engineering Research Center of Advanced Functional Material Manufacturing of Ministry of Education; School of Chemical Engineering, Zhengzhou University, Zhengzhou 450001, China*

[2] *School of Mechanical and Power Engineering, Zhengzhou University, Zhengzhou, 450001, China*

[*] Corresponding author: jinyouzh@zzu.edu.cn (J.Y. Z.).

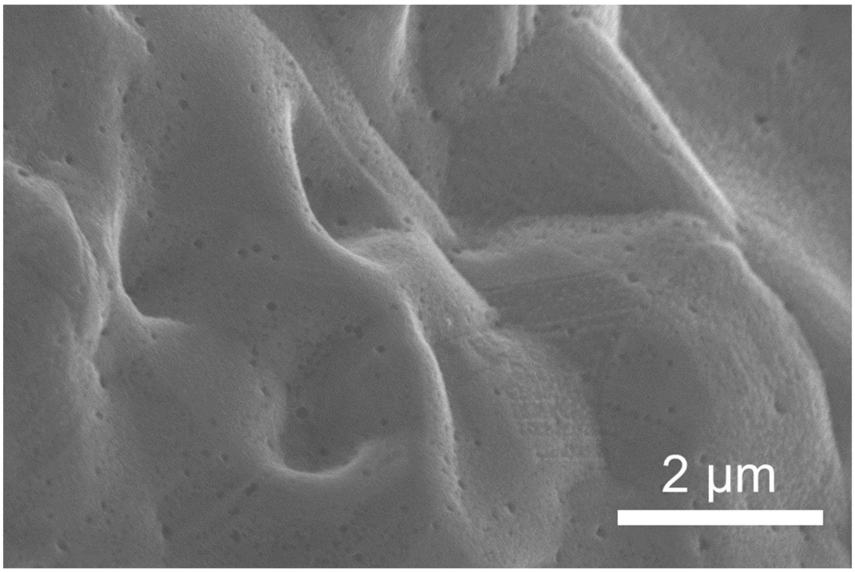

Figure S1. SEM image of the bare Co foam.

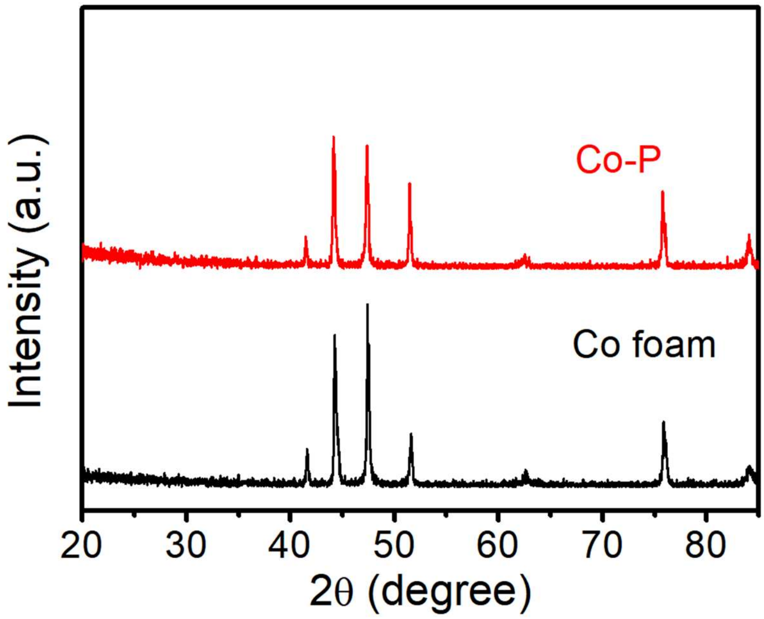

Figure S2. XRD patterns of Co-P and Co foam.

Co-P was prepared by phosphorization of the Co foam in tube furnace. 1.0 g $NaH_2PO_2{\cdot}H_2O$ and Co foam was separately put into quartz tube with two ceramic boats, $NaH_2PO_2{\cdot}H_2O$ was located in the upstream. The quartz tube was flowed by Ar gas for 30 min and then kept at 300 °C for 1 h (heating rate 5 °C/min) with Ar atmosphere. The Co-P was finally obtained after naturally cooling down to room temperature.

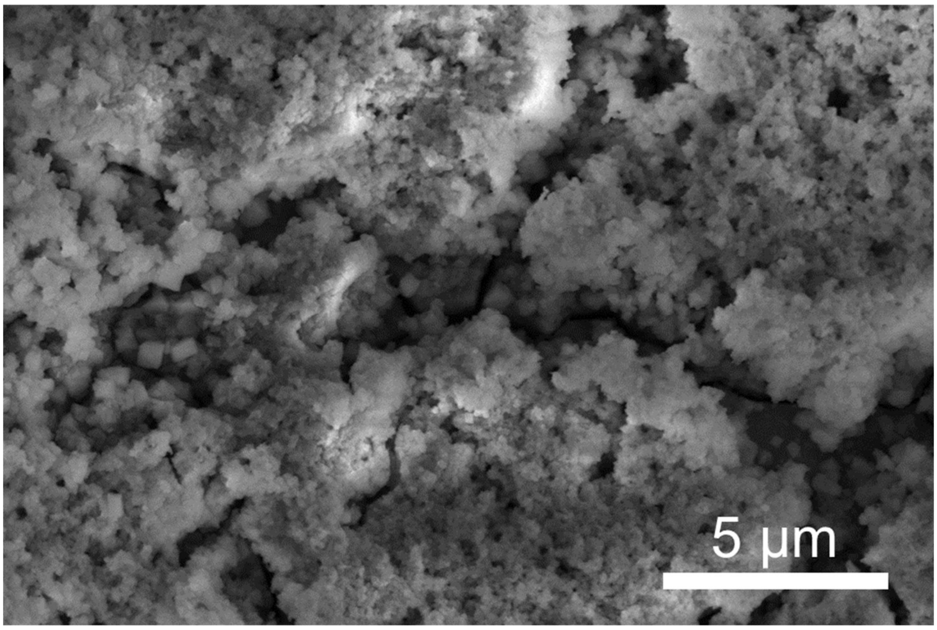

Figure S3. SEM image of NiFe-CoP/CF.

NiFe-CoP/CF was prepared by the following steps. CoP/CF was submerged in a solution containing 0.05 M $NiCl_2$ and 0.05 M $FeCl_3$, and then it was removed and heated at 80 °C for 4 h. After one more round of submerging and heating, NiFe-CoP/CF was obtained without phosphorization.

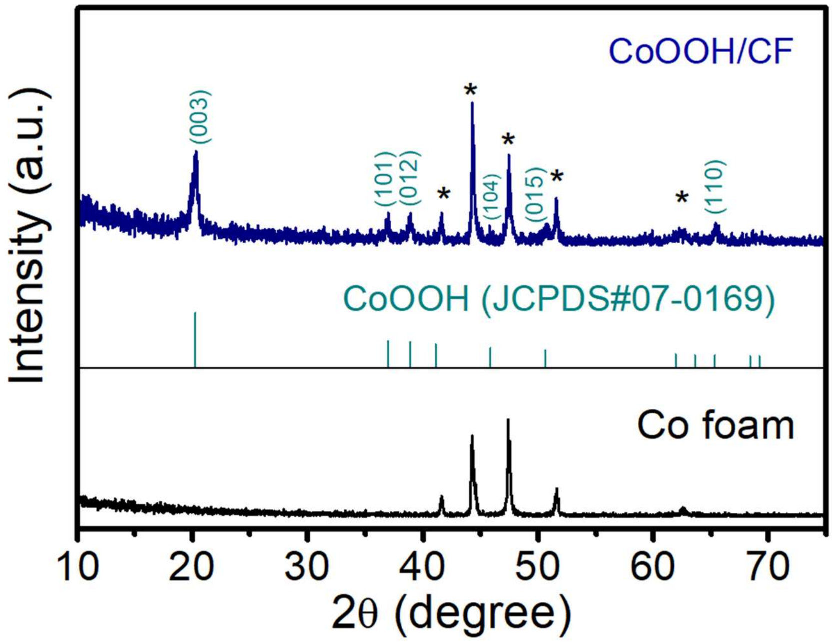

Figure S4. XRD patterns of CoOOH/CF and Co foam.

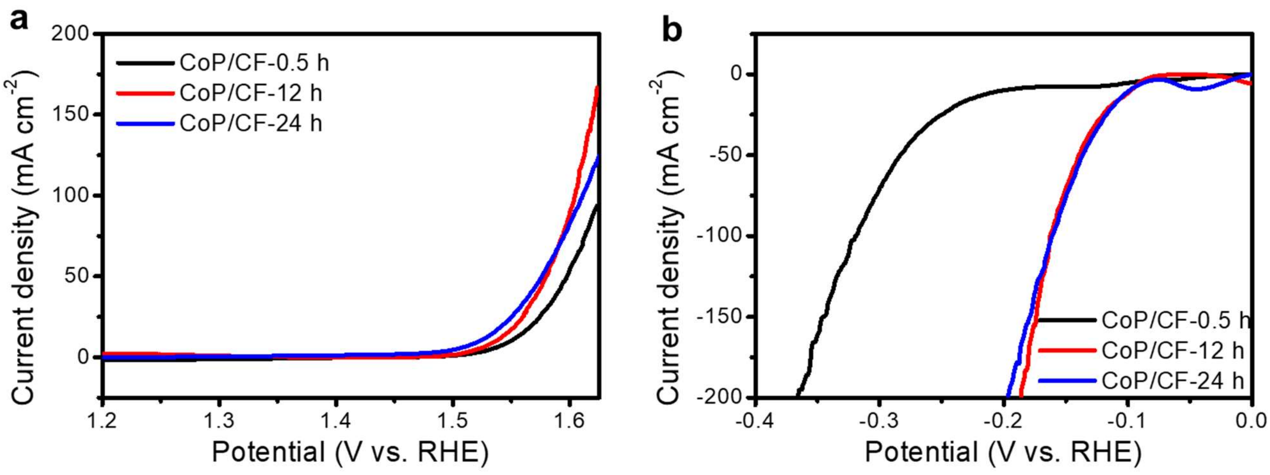

Figure S5. (a) OER and (b) HER polarization curves of the different CoP/CF electrodes obtained from CoOOH with different growing times of 0.5 h, 12 h, and 24 h in 4 M NaOH solution.

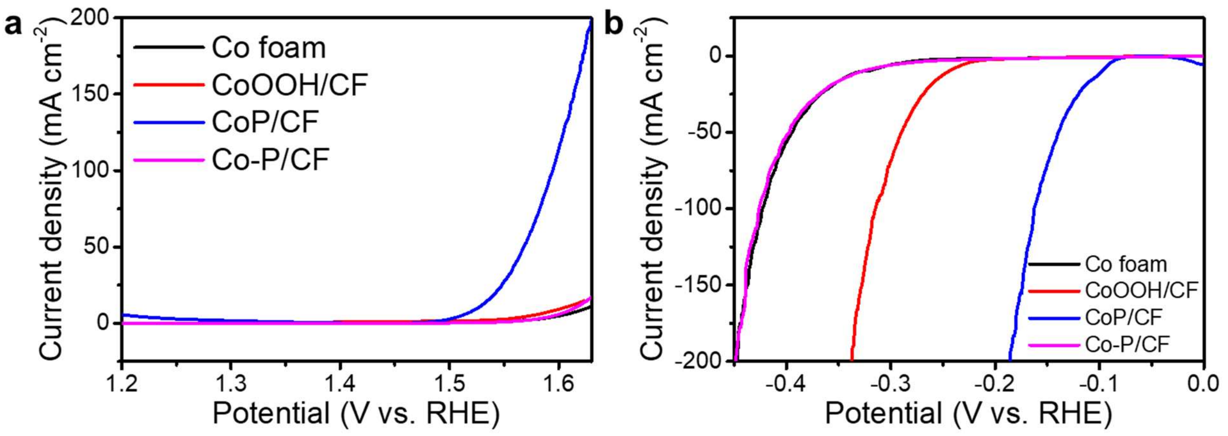

Figure S6. (a) OER and (b) HER polarization curves of Co foam, CoOOH/CF, CoP/CF, and Co-P/CF in 1.0 M KOH solution.

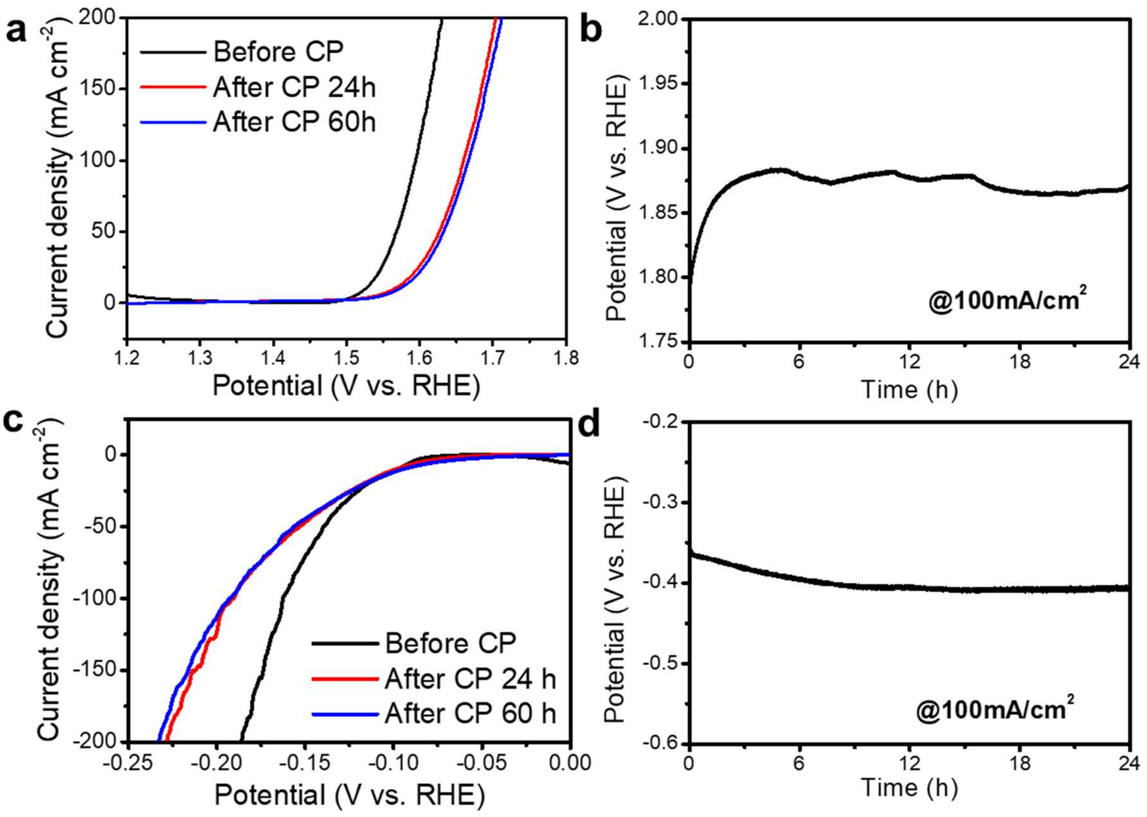

Figure S7. Long-term tests of CoP/CF at 100 mA/cm^2. (a) OER and (c) HER polarization curves of CoP/CF, (b) and (d) chronopotentiometry (CP) measurements of OER and HER at a current density of 100 mA/cm^2 for 24 h.

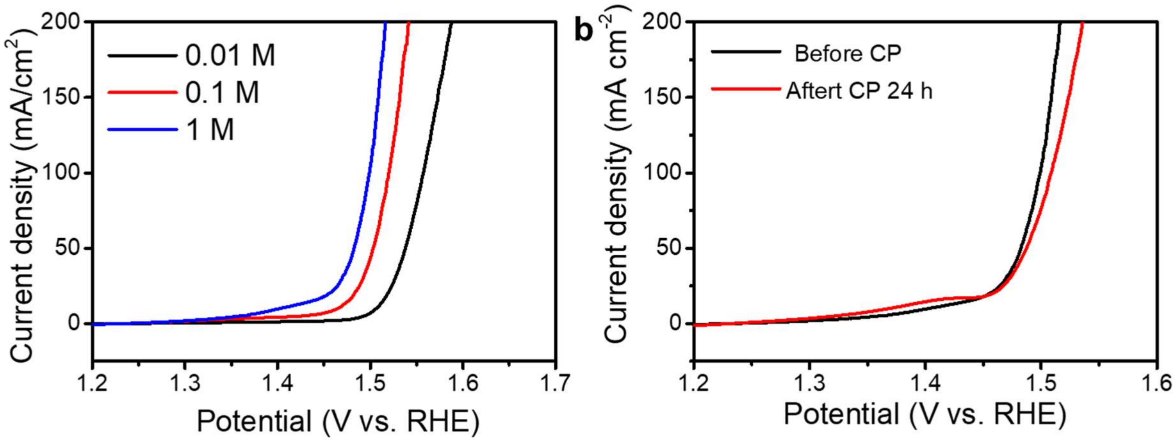

Figure S8. (a) OER polarization curves of NiFeP-CoP/CF electrodes obtained from immerging in a solution containing 0.01 M (0.005 M $NiCl_2$ and 0.005 M $FeCl_3$), 0.1 M (0.05 M $NiCl_2$ and 0.05 M $FeCl_3$), and 1.0 M (0.5 M $NiCl_2$ and 0.5 M $FeCl_3$) mixed Fe^{3+} and Ni^{2+}. (b) OER polarization curves of NiFeP-CoP/CF obtained from immerging in a solution containing 1.0 M (0.5 M $NiCl_2$ and 0.5 M $FeCl_3$) mixed Fe^{3+} and Ni^{2+} solution before and after CP measurement at 100 mA/cm^2.

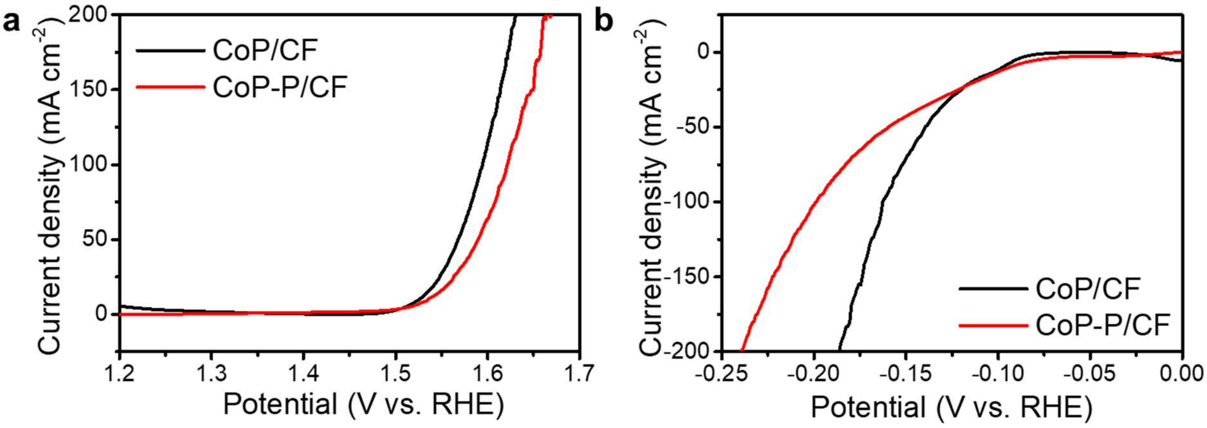

Figure S9. (a) OER and (c) HER polarization curves of CoP/CF, CoP-P/CF (phosphorization of CoP/CF).

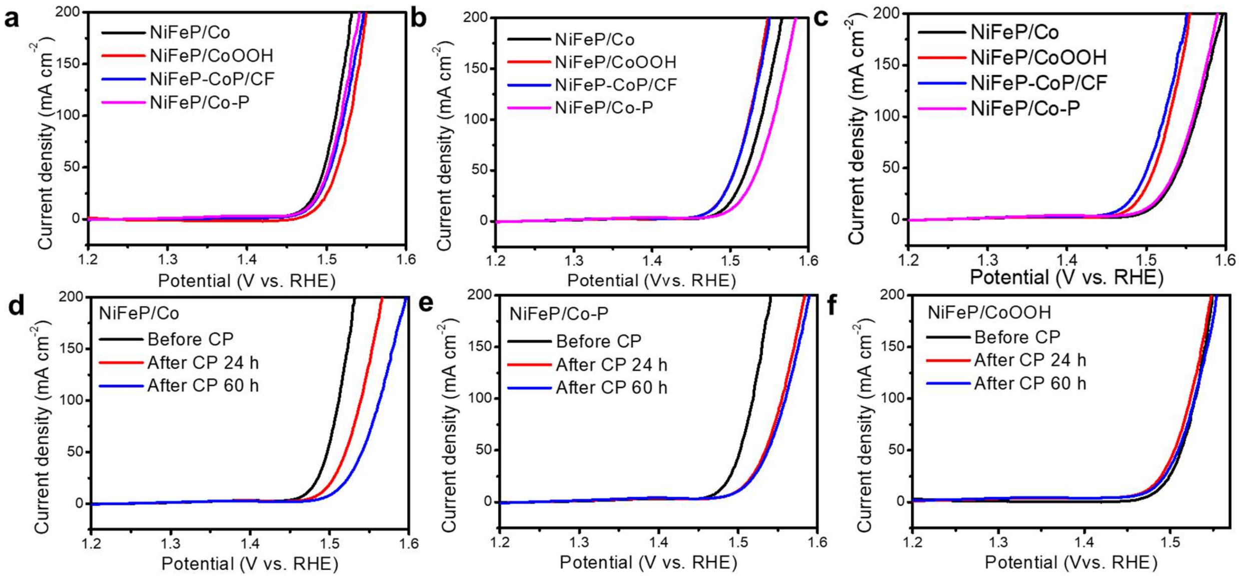

Figure S10. Long-term OER stability testing of NiFeP/Co, NiFeP/CoOOH, NiFeP-CoP/CF, and NiFeP/Co-P at 100 mA/cm^2 for 24 h and 60 h. (a) Polarization curves before chronopotentiometry measurement, (b) Polarization curves after CP for 24 h, (c) Polarization curves after chronopotentiometry measurement for 60 h, (d) LSV of NiFeP/Co, (e) LSV of NiFeP/Co-P, (f) Polarization curves of NiFeP/CoOOH.

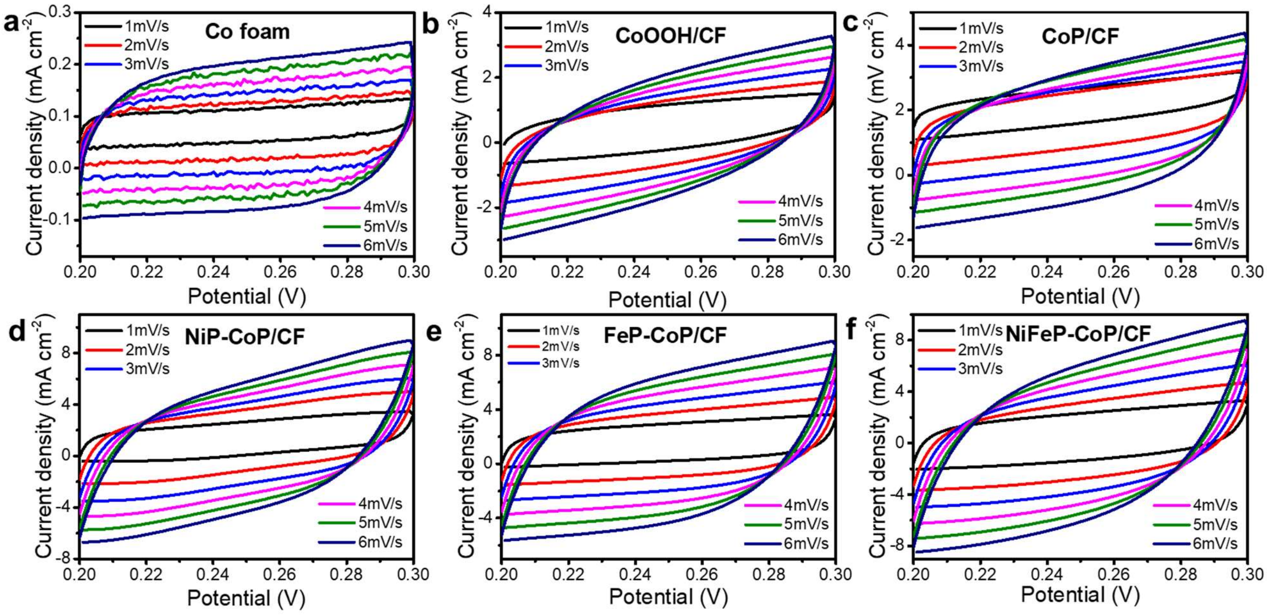

Figure S11. CV curves showing the capacitive of electrochemical double layer of (a) Co foam, (b) CoOOH/CF, (c) CoP/CF, (d) NiP-CoP/CF, (e) FeP-CoP/CF, and (f) NiFeP-CoP/CF for OER.

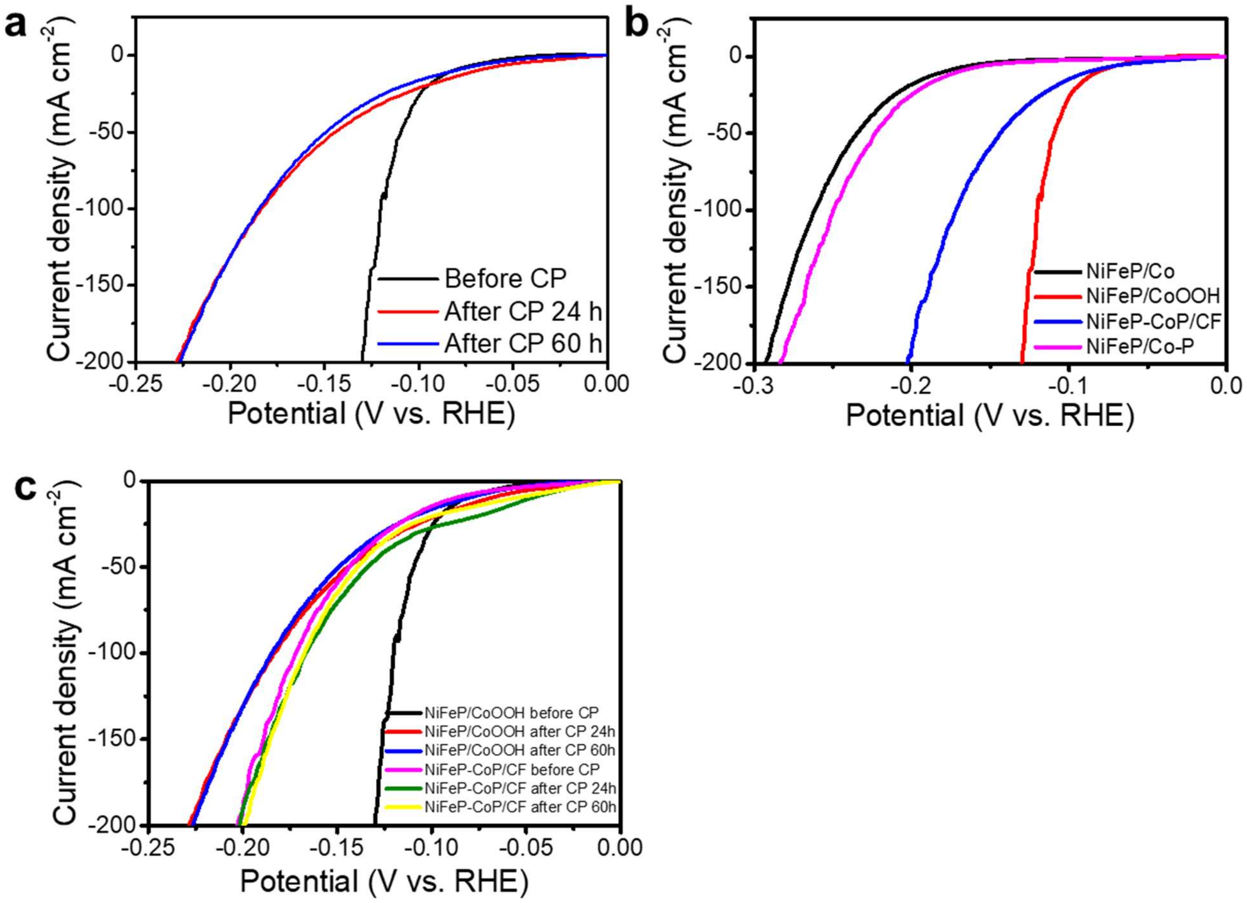

Figure S12. Long-term HER stability testing at 100 mA/cm^2 for 24 h and 60 h. HER Polarization curves of (a) NiFeP/CoOOH, (b) after CP measurement, (c) before and after CP measurements.

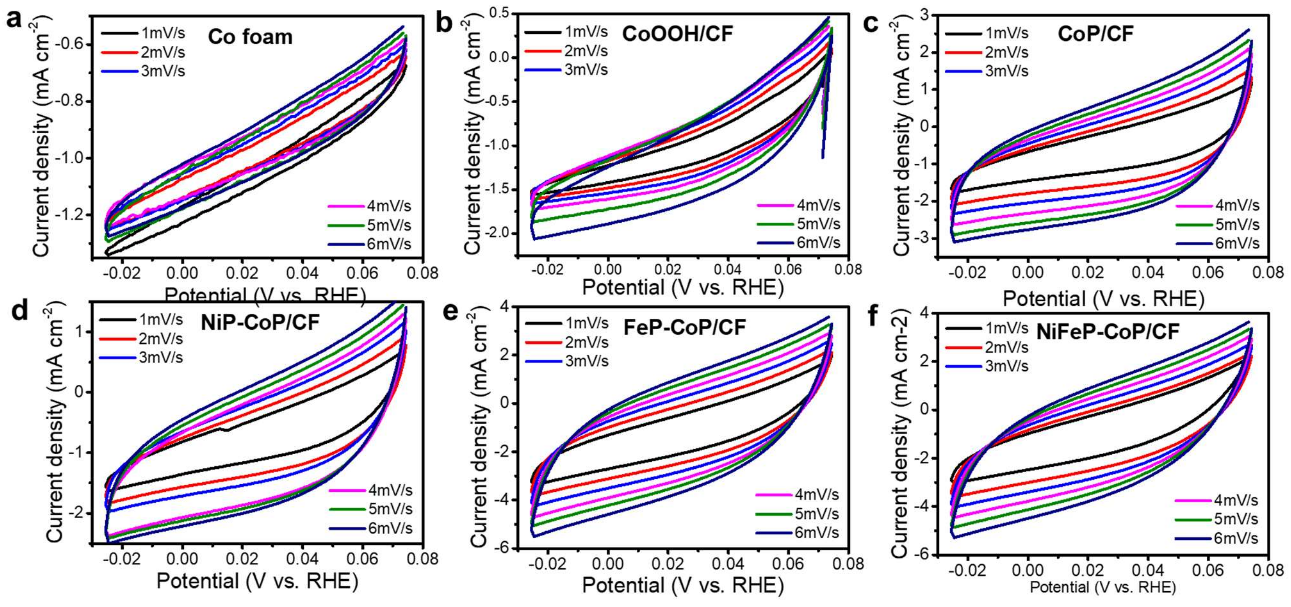

Figure S13. CV curves showing the capacitive of electrochemical double layer of (a) Co foam, (b) CoOOH/CF, (c) CoP/CF, (d) NiP-CoP/CF, (e) FeP-CoP/CF, and (f) NiFeP-CoP/CF for HER.

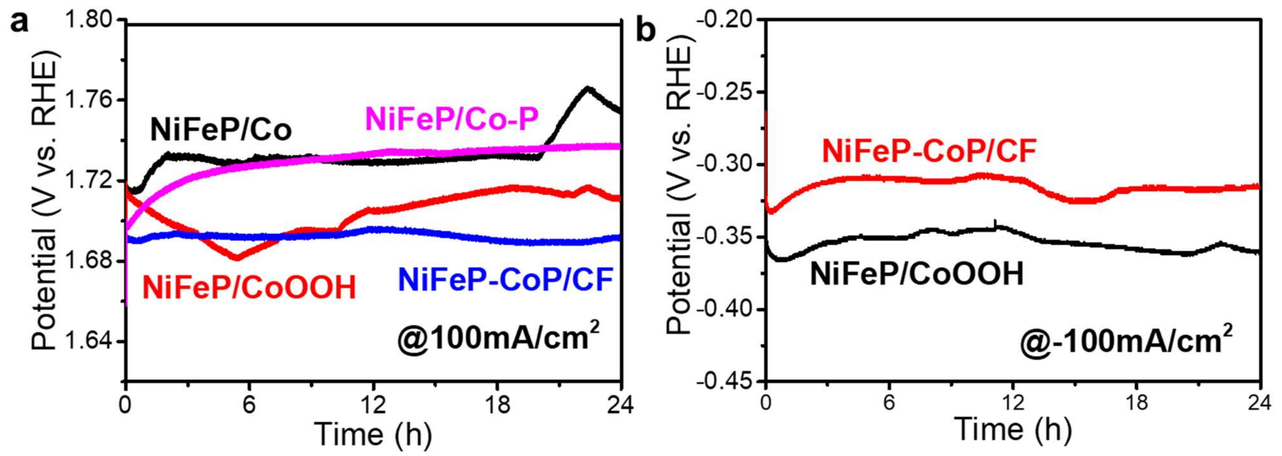

Figure S14. Long-term (a) OER and (b) HER stability testing by chronopotentiometry measurement at 100 mA/cm^2 for 24 h.

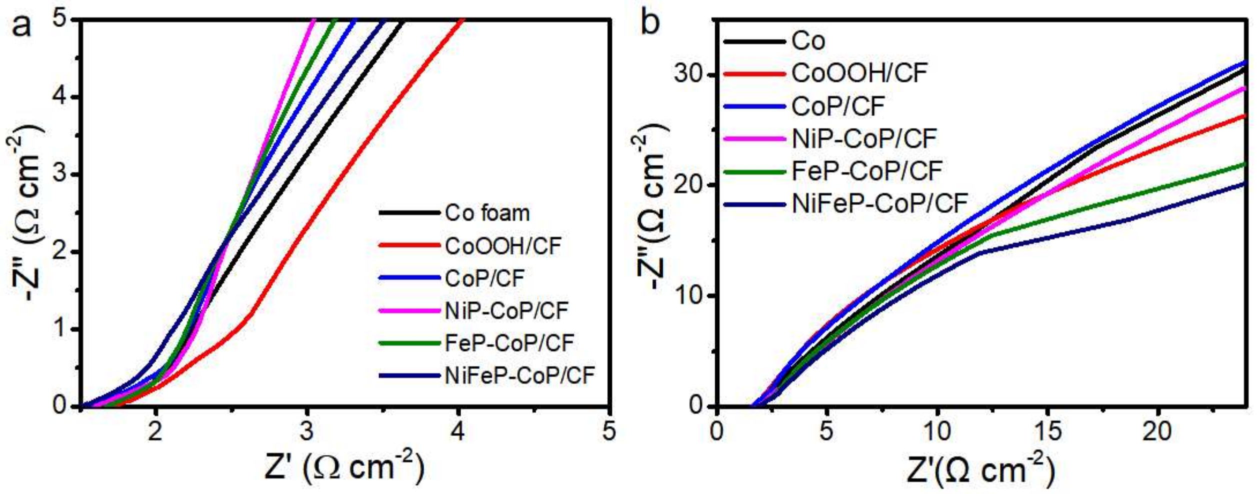

Figure S15. Electrochemical impedance spectroscopy (EIS) of electrodes were testing with 0.01 Hz – 100 kHz for (a) OER, and (b) HER.

Table S1. The overpotentials of different samples for OER.

Samples	η_{10}/mV	η_{20}/mV	η_{50}/mV	η_{100}/mV
Co foam	396			
CoOOH/CF	373			
CoP/CF	295	312	337	367
NiP-CoP/CF	295	317	349	370
FeP-CoP/CF	256	271	291	308
NiFeP-CoP/CF	245	257	275	292
NiFeP-CoP/CF after CP 24h	244	257	274	292
NiFeP-CoP/CF after CP 60h	237	251	271	291

Table S2. The overpotentials of different samples for HER.

Samples	η_{-10}/mV	η_{-20}/mV	η_{-50}/mV	η_{-100}/mV
Co foam	325	359	396	422
CoOOH/CF	245	262	290	315
CoP/CF	98	115	139	163
NiP-CoP/CF	109	123	149	170
FeP-CoP/CF	104	123	150	176
NiFeP-CoP/CF	89	110	144	172
NiFeP-CoP/CF after CP 24h	47	74	134	166
NiFeP-CoP/CF after CP 60h	56	98	139	167
